# Ankle–Brachial Index and Lifestyle Factors Among Women of Reproductive and Postmenopausal Age: A Cross-Sectional Study from Primary Care Settings in Croatia

**DOI:** 10.3390/jcm14238286

**Published:** 2025-11-21

**Authors:** Ema Dejhalla, David Zahirović, Juraj Sinožić, Tina Zavidić, Karmela Bonassin, Nensi Bilanović Ćoso, Tamara Sinožić

**Affiliations:** 1 Family Medicine Office, Medical Centre for Occupational Health Rijeka, Verdieva 8, 51000 Rijeka, Croatia; 2 Faculty of Medicine, University of Rijeka, Braće Branchetta 20, 51000 Rijeka, Croatia; david.zahirovic@medri.uniri.hr (D.Z.); jurajsinozic8@gmail.com (J.S.); tina.zavidic@medri.uniri.hr (T.Z.); bilanovicnensi@yahoo.com (N.B.Ć.); tamara.sinozic@medri.uniri.hr (T.S.); 3 Community Health Centre of the Primorje-Gorski Kotar County, Krešimirova 52a, 51000 Rijeka, Croatia; 4 Istrian Health Centres, Lupoglav 10B, 52100 Lupoglav, Croatia; 5 Family Medicine Office Karmela Bonassin, 9. rujna 1, 52341 Žminj, Croatia; ordinacija.bonassin@gmail.com; 6 Family Medicine Office Nensi Bilanović Ćoso, Dražice bb, 51218 Dražice, Croatia; 7 Family Medicine Office Tamara Sinožić, Barba Rike 5a, 51417 Mošćenička Draga, Croatia

**Keywords:** ankle brachial index, life style, peripheral arterial disease, postmenopause, reproduction

## Abstract

**Background/Objectives:** Cardiovascular diseases remain the leading cause of death among women, with peripheral arterial disease (PAD) representing an important manifestation of systemic atherosclerosis. The ankle–brachial index (ABI) is a simple, non-invasive measure used for PAD screening and cardiovascular risk assessment. This study aimed to compare ABI values between women of reproductive and postmenopausal age and to explore associations with lifestyle and clinical characteristics in primary care settings. **Methods:** This cross-sectional study included 437 women recruited from family medicine offices in two Croatian counties between November and December 2024. Participants completed validated questionnaires on dietary habits (MEDAS) and physical activity (IPAQ/PASE), and underwent anthropometric and blood pressure measurements. ABI was assessed using an automated MESI device following a standardized protocol. Multivariable logistic regression was performed to identify independent predictors of abnormal ABI (<1.00), adjusting for age, BMI, hypertension, smoking, and county. **Results**: Most participants had normal ABI values (right 95.7%; left 95.0%). Mild to moderate PAD (ABI 0.90–0.99) was observed in approximately 3% and severe PAD (ABI < 0.90) in ≤1.1% of cases. Postmenopausal women were more likely to present with lower ABI values (*p* = 0.046), though this association was attenuated after adjusting for age. Lifestyle factors, including diet and physical activity, showed no significant association with ABI in the adjusted models. **Conclusions**: Age emerged as the primary determinant of reduced ABI, while hypertension and smoking showed positive but non-significant associations. The findings underscore the importance of early vascular screening and lifestyle-based prevention in women, particularly in the postmenopausal period. Larger longitudinal studies are warranted to clarify causal pathways and the role of hormonal and behavioral factors in peripheral arterial disease development.

## 1. Introduction

Peripheral arterial disease (PAD) is a manifestation of systemic atherosclerosis that affects the arteries of the lower extremities and represents an important indicator of cardiovascular morbidity and mortality [1,2]. It develops due to atherosclerotic processes that reduce arterial elasticity and blood flow, leading to ischemic symptoms that range from mild claudication to advanced stages requiring amputation [3]. The global prevalence of PAD continues to rise, particularly among older adults and women, reflecting population aging and the growing burden of cardiovascular risk factors such as hypertension, diabetes, obesity, and smoking [4,5].

Early detection of impaired peripheral perfusion is essential because PAD often remains asymptomatic until advanced stages. The ankle–brachial index (ABI) is a simple, non-invasive, and widely accepted diagnostic tool used to assess arterial obstruction and identify individuals at risk for cardiovascular events [6,7]. ABI provides information on lower-extremity circulation as well as overall atherosclerotic burden and vascular health [8]. Values below 0.90 indicate peripheral arterial obstruction, while values above 1.40 suggest arterial stiffening due to medial calcification [9]. Numerous studies have demonstrated that abnormal ABI values are associated with increased risks of coronary heart disease, stroke, and cardiovascular death [10,11,12].

Despite substantial progress in understanding PAD pathophysiology, sex-specific and age-related differences in vascular health remain underexplored. Menopause-related estrogen decline is associated with endothelial dysfunction, arterial stiffness, and increased prevalence of hypertension and dyslipidemia [13,14]. These physiological alterations may contribute to a greater burden of PAD among postmenopausal women. However, the relationship between menopausal status, lifestyle habits, and peripheral arterial health remains insufficiently investigated, especially in Central and Southeastern Europe, where regional and sociodemographic variations may influence cardiovascular risk patterns [15,16,17].

Lifestyle factors play a crucial role in the prevention and progression of PAD. Adherence to a Mediterranean diet, rich in fruits, vegetables, whole grains, fish, and olive oil, has been associated with improved endothelial function, reduced inflammation, and lower cardiovascular mortality [18,19]. Regular physical activity also contributes to better vascular perfusion, improved metabolic control, and decreased arterial stiffness [20,21]. Conversely, smoking remains one of the most significant modifiable risk factors for PAD, accelerating atherosclerotic changes and impairing endothelial repair mechanisms [22].

Given these considerations, this study aimed to evaluate the relationship between cardiovascular health, lifestyle factors, and sociodemographic characteristics among women from Primorje–Gorski Kotar and Istria Counties in Croatia, with a particular focus on differences between women of reproductive age and postmenopausal women. Special emphasis was placed on examining the association between ABI values and cardiovascular risk profiles, as well as potential regional variations in vascular health, dietary habits, and physical activity. The findings are expected to contribute to a better understanding of the interplay between aging, menopause, and modifiable lifestyle factors in the early identification and prevention of peripheral arterial disease in women.

## 2. Materials and Methods

### 2.1. Study Design and Population

A cross-sectional study was conducted from November 2024 to December 2024. It was carried out in six family medicine offices in Primorje-Gorski Kotar County, located in Rijeka, Dražice, and Mošćenička Draga, and in three offices in Istria County, located in Lupoglav, Pula, and Žminj.

Four offices in Primorje-Gorski Kotar County operate as independent units with individual practitioners, one is part of a healthcare institution, and one is a private practice. In Istria County, one office (in Lupoglav) is part of the Istrian Health Centers, while the other two (Pula and Žminj) are independent units with individual practitioners.

The target sample size for this study was determined a priori using a standard formula for estimating prevalence in a finite population [9]. Population data for Primorje–Gorski Kotar County and Istria County were used to define the sampling frame. In Primorje–Gorski Kotar County there are 137,644 women in total, of whom 68,200 are of reproductive age and 69,300 are older. In Istria County there are 101,125 women in total, of whom 51,500 are of reproductive age and 49,556 are older [10]. For the purpose of sample size calculation, women of reproductive age and postmenopausal women were considered separately. The combined population of women of reproductive age across both counties was therefore 119,700, and the combined postmenopausal/older female population was 118,856.

Published estimates indicate that peripheral arterial disease (PAD) affects approximately 12% of women of reproductive age and 30% of postmenopausal women [11]. These expected prevalences (p), together with the corresponding source population sizes (N), were entered into the sample size formula to estimate the minimum number of participants needed in each stratum to estimate PAD prevalence with acceptable precision. This yielded required sample sizes of 163 women of reproductive age and 246 postmenopausal women across both counties.

**Figure 1:**
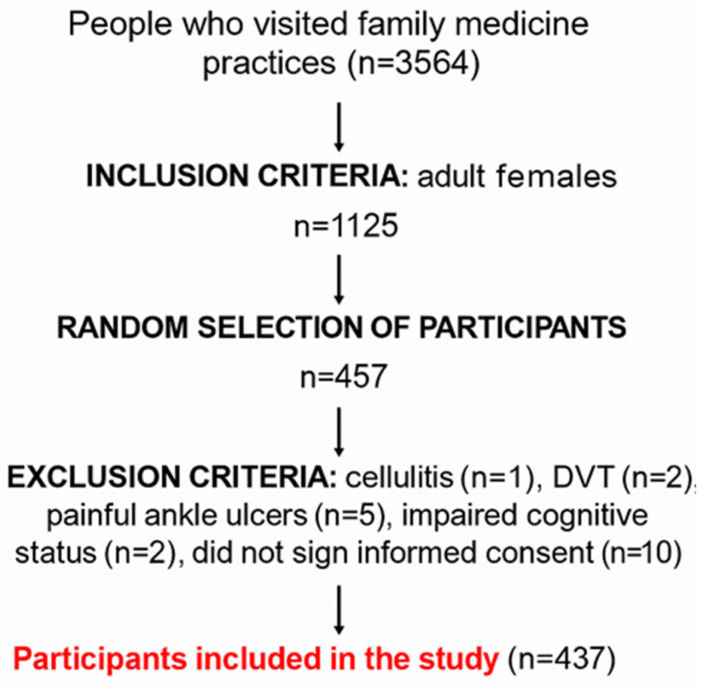
Participant recruitment.

These target numbers (163 premenopausal/reproductive-age women and 246 postmenopausal women) were used as the planned recruitment goals for the study. The achieved sample was then compared with these targets to assess whether each stratum was adequately represented in the final analysis set. The study sample was randomized. It was determined that a minimum of 19 women of reproductive age and 28 postmenopausal women needed to be recruited from each of the nine selected offices. Women attending check-ups at the selected offices were randomly included in the study. Inclusion criteria required participants to be female and at least 18 years old. Exclusion criteria included contraindications for ABI measurement, such as cellulitis, deep vein thrombosis, painful ulcers on the ankle, and cognitive impairment, as these conditions would prevent participants from completing the questionnaire. Figure 1 shows the flowchart of participant recruitment.

A total of 437 participants took part in the study. Each participant was surveyed and examined by the principal investigator (15 min per participant) in the office where they received regular care. Before the survey began, the purpose of the study was explained in detail, participation was confirmed as voluntary, and anonymity was guaranteed. Participants who agreed to take part signed an informed consent form and then completed a written questionnaire.

#### Ethical Considerations

The study adhered to bioethical standards, including the four fundamental bioethical principles (personal integrity–autonomy, justice, beneficence, and non-maleficence), as well as ethical guidelines derived from them, in accordance with the Nuremberg Code, the latest revision of the Helsinki Declaration, and other relevant documents. The Ethics Committee for Biomedical Research of the Faculty of Medicine, University of Rijeka, approved the study at its second session, held electronically on 19 November 2024 (CLASS: 007-08/24-01/82, REF. NO.: 2170-1-42-04-3/1-24-4).

### 2.2. Data Collection

#### 2.2.1. Questionnaires

Sociodemographic data (age, education, employment status, place of residence), medical history (personal medical history, menstrual status, chronic therapy, smoking), and lifestyle habits were collected for all participants.

All participants completed the validated MEDAS questionnaire on adherence to the Mediterranean diet [12]. The MEDAS questionnaire consists of 14 questions covering various aspects of the Mediterranean diet, including the intake of specific food items and cooking methods. Each answer was scored 0 or 1, depending on whether the habit aligned with Mediterranean diet recommendations. The 14-item MEDAS questionnaire has undergone validation in several European countries, consistently demonstrating strong reliability and validity in assessing adherence to the Mediterranean diet. Research comparing MEDAS scores with detailed dietary records and food frequency questionnaires has reported moderate to high correlations (ICC values generally between 0.69 and 0.80), confirming its effectiveness in capturing actual dietary intake. Agreement at the individual item level is particularly robust for essential dietary components such as olive oil, fruits, and vegetables [13].

Its stable psychometric properties across diverse populations emphasize MEDAS as a reliable and versatile instrument for nutritional evaluation. Owing to its simplicity and time efficiency, it is particularly well-suited for large-scale epidemiological studies and public health programs, where resources may be limited.

In conclusion, MEDAS is a scientifically supported, reliable, and user-friendly tool for monitoring adherence to the Mediterranean diet, making it highly applicable in both clinical research and population-level health surveillance throughout Europe. A score of 9–14 points indicates strong adherence, reflecting a dietary pattern rich in fruits, vegetables, legumes, whole grains, fish, and healthy fats such as olive oil, while limiting red meat and processed foods. Scores between 5 and 8 points represent moderate adherence, suggesting partial compliance with Mediterranean dietary principles and room for improvement through increased consumption of beneficial foods. Scores from 0 to 4 points indicate low adherence, meaning the individual’s eating habits diverge considerably from the Mediterranean dietary pattern [13,14,15,16,17].

Participants aged 18 to 69 completed the validated IPAQ questionnaire on physical activity [18]. The IPAQ questionnaire gathers data on how many days per week and how much time per day individuals spend engaging in various physical activities, including vigorous exercise (e.g., aerobics, heavy lifting), moderate exercise (e.g., cycling at a steady pace, carrying light loads), walking, and sedentary behavior such as sitting. For each activity type, respondents report frequency (days per week) and duration (minutes per day). To estimate overall energy expenditure, activities are assigned standardized metabolic equivalent of task (MET) values: vigorous activity = 8.0 METs, moderate activity = 4.0 METs, and walking = 3.3 METs.

A high level of physical activity is defined by meeting one of the following thresholds: performing vigorous activity on at least 3 days and accumulating a total of 1500 MET-minutes per week, or engaging in any combination of walking, moderate, and vigorous activities over 7 or more days that reaches at least 3000 MET-minutes per week [19,20,21,22].

Participants aged 70 and older completed the validated PASE questionnaire (Physical Activity Scale for the Elderly) [23]. The PASE questionnaire quantifies physical activity over the past week, covering household activities, recreational activities, and occupational tasks. It considers both frequency and intensity to provide a comprehensive assessment of physical activity levels in older adults [24].

#### 2.2.2. Anthropometric Measurements and Medical Examination

Anthropometric measurements, including height and weight were obtained using International Society for the Advancement of Kinanthropometry (ISAK) protocols. Body mass index (BMI) was determined as weight in kilograms divided by height in meters squared. The same equipment was used for all participants, and all measurements were performed by the same trained professional. Body weight was measured using a calibrated digital floor scale (“Seca” 200 kg) with an accuracy of 100 g. Height was measured using a “Seca” stadiometer. During height measurements, participants stood upright with heels together and touching the stadiometer, while their head was positioned according to the Frankfurt horizontal plane—ensuring that the line connecting the lower edge of the eye socket and the upper edge of the tragus was parallel to the floor [11]. The nearest height value was recorded with an accuracy of 0.1 cm.

All ABI measurements were performed in a standardized manner by trained staff using an automated oscillometric device (MESI ABPI MD^®^, MESI d.o.o., Ljubljana, Slovenia), which simultaneously measures systolic pressures in both ankles and arms using four cuffs [25]. The protocol followed current recommendations for patient preparation and limb positioning to minimize known sources of measurement error. Patients were instructed to avoid nicotine prior to testing, because smoking can selectively elevate systolic pressure at the ankle without affecting brachial pressure and therefore artifactually increase the ABI. After arrival, each patient rested in a quiet examination room for 10–30 min to achieve hemodynamic stabilization. Measurements were taken with the patient lying flat in the fully supine position, with the head and all extremities supported and level with the right atrium. Sitting is not acceptable for ABI determination, because ankle systolic pressures obtained in the seated position can increase the calculated ABI by approximately 0.3 compared with the supine position, which can mask peripheral arterial disease [26]. The upper and lower limbs were supported to reduce voluntary or postural muscle activity, which improves patient relaxation and minimizes motion artefact during cuff inflation/deflation. Any open wounds were covered with a clean barrier dressing before cuff placement to reduce risk of wound or equipment contamination.

Appropriately sized occlusion cuffs were placed on the upper arms (brachial artery) and just above each ankle (posterior tibial/dorsalis pedis level) according to manufacturer instructions. For each limb, cuff width was selected to cover at least 40% of limb circumference and cuff bladder length to cover at least 80% of the limb circumference, to avoid overestimation with cuffs that are too narrow or underestimation with cuffs that are too wide. The MESI ABPI MD device automatically inflated and deflated all cuffs and detected systolic pressures oscillometrically. For each leg, ABI was calculated as the ratio of ankle systolic pressure to brachial systolic pressure. Consistent with guideline practice, the higher of the two ankle artery systolic pressures (posterior tibial or dorsalis pedis) in a given leg was divided by the highest systolic brachial pressure recorded in either arm to obtain that leg’s ABI. If an arm pressure was clearly physiologically unreliable (e.g., >10–15 mmHg lower on one side suggesting subclavian stenosis), the higher brachial pressure was used as reference [27].

An ABI between 0.90 and 1.29 was classified as normal perfusion, while ABI < 0.90 was considered diagnostic of peripheral arterial disease (PAD). Values >1.40 were classified as noncompressible/calcified arteries and therefore non-interpretable as a measure of perfusion; in those cases the ABI result was not used for PAD severity grading, and the limb was flagged as having medial arterial calcification rather than normal flow. When an ABI result was <0.90, >1.40, or when cuff inflation was affected by patient movement or arrhythmia (device error message or obviously inconsistent pressures), the entire measurement sequence was repeated after an additional brief rest in the same supine position. The final ABI for analysis was the repeatable value.

The MESI ABPI MD system has been validated against the Doppler gold-standard method in multiple studies. In primary care screening, MESI-derived ABI showed good agreement with Doppler ABI, with a small mean positive bias of about 0.06 ± 0.14 and strong correlation (R ≈ 0.61, *p* < 0.0001); using an ABI_mesi < 1.0 to detect Doppler ABI < 0.9 (peripheral arterial disease) yielded a sensitivity of 85% and specificity of 96%, and measurements were completed roughly three times faster than Doppler [28].

These data support the feasibility of MESI for rapid standardized ABI acquisition at the bedside, while highlighting that extreme ABI values (particularly very low or very high) require cautious interpretation and, when clinically relevant, confirmation with repeat measurement or adjunct vascular testing.

To minimize potential measurement bias, the investigator performing ABI assessments was blinded to participants’ questionnaire responses, lifestyle data and medical history whenever feasible. Questionnaire forms and clinical information were not visible during ABI acquisition, and measurements were conducted following a standardized protocol without reference to individual clinical characteristics.

### 2.3. PAD Diagnosis

The classification of ABI measurement results was presented in Table 1 [1].

### 2.4. Statistical Analysis

For statistical analysis, both descriptive and inferential statistical methods were used. Descriptive analysis presented data in tabular form as absolute frequencies and percentages, as well as graphically using diagrams. Testing was conducted using the χ^2^ test. The significance level for all tests was set at 5%, corresponding to a 95% confidence level. Based on the obtained significance values, decisions were made regarding the acceptance or rejection of the hypotheses, with all *p*-values reported as two-sided.

In addition to descriptive statistics and χ^2^ tests, multivariable regression modeling was used to estimate the independent associations between clinical and lifestyle factors and peripheral arterial disease (PAD). PAD was defined dichotomously as ABI < 0.90 in at least one leg versus ABI ≥ 0.90 and ≤ 1.40. Participants with ABI > 1.40 (indicating non-compressible/calcified arteries) were excluded from the PAD vs no-PAD comparisons because such values reflect arterial stiffness rather than hemodynamically relevant obstruction.

Binary logistic regression models with PAD (yes/no) as the dependent variable We constructed. The primary exposure of interest was menopausal status (postmenopausal vs. reproductive age). Covariates were selected a priori based on biological plausibility and known links to lower-extremity atherosclerosis: age (continuous, in years), body mass index (BMI, kg/m^2^, continuous), hypertension (yes/no, defined as systolic BP ≥ 140 mmHg and/or diastolic BP ≥ 90 mmHg or current antihypertensive therapy), and county (Primorje–Gorski Kotar vs. Istria) to account for regional differences in age structure, smoking, and blood pressure distribution. Smoking status (current vs. non-smoker) was then added in a second step as a cardiovascular risk factor. Adjusted odds ratios (aORs) with 95% confidence intervals (95% CIs) are reported.

To explore vascular health more broadly (and increase statistical power), sensitivity models were also run using “any ABI abnormality” as the dependent variable. In this analysis, the outcome combined borderline ABI (0.90–0.99), mild PAD (0.80–0.89), moderate PAD (0.50–0.79), and severe PAD (<0.50) into a single abnormal ABI category, contrasted against normal ABI (1.00–1.40). The same covariates (age, BMI, hypertension, county, smoking) were included. This approach captures early hemodynamic impairment rather than only clinically manifest PAD and mitigates sparse-cell problems.

Because lifestyle was a focus of the study, additional multivariable models examined whether higher physical activity or higher Mediterranean diet adherence were independently associated with abnormal ABI. For women <70 years, physical activity level as defined by IPAQ (high vs. moderate/low) was included as an exposure. For women ≥70 years, PASE activity category (high vs. low/moderate) was used. Mediterranean diet adherence (high vs. moderate/low, based on MEDAS score ≥ 9) was also modeled. Each of these lifestyle variables was entered separately with the same adjustment set (age, BMI, hypertension, county, smoking, menopausal status). Results are presented as aORs with 95% CIs.

Collinearity was assessed by examining standard errors and variance inflation factors; no instability suggesting problematic collinearity between menopausal status and chronological age was observed in the final models, but age remained in all models regardless of statistical significance due to its clinical relevance. Model fit was assessed with the Hosmer–Lemeshow test.

Given that the observed prevalence of PAD (ABI < 0.90) in this sample was low (approximately 3–4%), post hoc power considerations were performed. With 437 women and an overall PAD prevalence of ~3%, the number of PAD events was on the order of 13–15 cases. Under these conditions, the study had adequate power (>80%) to detect large effects (odds ratios around 3.0–3.5 or higher) for common exposures such as menopausal status or hypertension but was underpowered to detect more modest effects (odds ratios ~1.5–2.0). Therefore, non-significant associations in the regression models should be interpreted cautiously as “inconclusive” rather than evidence of no association. Minimum detectable effect sizes for exposures with ≥40% prevalence (e.g., postmenopausal status, hypertension) were in the range of OR ≈3 at α = 0.05, two-sided, whereas rarer exposures require even larger effects to reach statistical significance with the available number of events.

All analyses were conducted in IBM SPSS Statistics Version 26.0 (IBM Corp., Armonk, NY, USA). Statistical significance was defined as two-sided *p* < 0.05, but for rare-event models we emphasize effect size estimates and confidence intervals rather than *p*-values alone.

## 3. Results

### 3.1. Participant Characteristics

Table 2 presents data on age, BMI, menstrual status, education, employment status, and place of residence.

The participants are divided into six age groups (0–29, 30–40, 41–50, 51–60, 61–70, 71+). BMI is categorized into four groups: underweight, normal weight, overweight and obesity.

Regarding menstrual status, participants are classified into those who have menstruation and those who do not. Education level is divided into four categories: lower, secondary, higher, and university education. Based on employment status, participants are categorized as employed, unemployed, or retired. Regarding place of residence, participants live in either urban or rural areas.

The study included 437 female participants, of whom the majority were over 60 years old (47.1%), while younger age groups were less represented. Regarding education, most participants had a secondary level of education (53.8%), while 22.7% were highly educated. Employment status showed that 46.5% of participants were employed, while 45.5% were retired. Furthermore, 54.7% of participants lived in rural areas, while 45.3% were from urban areas. The BMI analysis revealed that 44.9% of participants had a normal weight, 35.9% were overweight, 17.6% were obese, and 1.6% were underweight. The participants were divided into two groups based on whether they had menstruation: women of reproductive age (39.4% of participants) and postmenopausal women (60.6% of participants). When examining the significance level for the questions on age, BMI, menstruation, education, employment status, and place of residence, it was observed that the significance value of the χ^2^ test was *p* < 0.05, indicating a statistically significant difference between the observed counties.

There was a statistically significant difference in the age of participants between Primorje-Gorski Kotar County and Istria County. In Istria County, there were more older participants (61+ years), while in Primorje-Gorski Kotar County, there were more younger participants. A difference in body mass was observed between the counties. Primorje-Gorski Kotar County had a higher percentage of participants with a normal body weight, while Istria County had a higher percentage of obese participants. There was a difference in the percentage of participants who still have menstruation—a higher percentage in Primorje-Gorski Kotar County compared to Istria County. Primorje-Gorski Kotar County had a higher percentage of highly educated participants compared to Istria County, where there was a higher proportion of people with secondary and lower education levels. In Primorje-Gorski Kotar County, more participants were employed, while in Istria County, more were retired. In Primorje-Gorski Kotar Countym, participants were mostly from urban areas, while in Istria County, participants were mostly from rural areas.

### 3.2. Chronic Diseases and Medication Use

Furthermore, in Table 3 and Table 4, the responses of participants to the question: chronic diseases and medications are shown.

Across the entire cohort, the presence of chronic diseases and the number or type of medications taken were not significantly associated with diet adherence (MEDAS), general physical activity (IPAQ), or physical activity among older participants (PASE). All comparisons showed non-significant *p*-values (chronic disease and MEDAS *p* = 0.147; medications and MEDAS *p* = 0.077; chronic disease and IPAQ *p* = 0.127; chronic disease and PASE *p* = 0.178; medications and IPAQ *p* = 0.145; medications and PASE *p* = 0.215).

### 3.3. Smoking, ABI, Blood Pressure, and Lifestyle Habits by County

In Table 5, a comparison of data on smoking, ankle-brachial index (ABI), blood pressure, and lifestyle habits is shown with respect to the observed counties.

Through statistical analysis, several significant differences were identified between Primorje-Gorski Kotar County and Istria County.

Smoking was significantly more prevalent in Primorje-Gorski Kotar County compared to Istria County (*p* = 0.001). Differences were also observed in blood pressure levels: participants from Istria County showed a higher prevalence of elevated systolic blood pressure (*p* < 0.001) and elevated diastolic blood pressure (*p* = 0.013). These findings indicate that, despite lower smoking rates, the Istrian sample had a greater burden of hypertension.

### 3.4. Mediterranean Diet Adherence (MEDAS)

When the association between adherence to the Mediterranean diet and sociodemographic or clinical variables was examined, no statistically significant differences were found overall (all *p* > 0.05). Variables including age (*p* = 0.133), BMI (*p* = 0.867), education (*p* = 0.200), employment (*p* = 0.133), smoking (*p* = 0.767), menstruation (*p* = 0.052), ABI on the right (*p* = 0.867) and left side (*p* = 0.634), as well as systolic (*p* = 0.155) and diastolic blood pressure (*p* = 0.199) were not associated with MEDAS scores when the total study population was analyzed. This suggests that, on a broad level, dietary adherence was not strongly differentiated by these demographic or health factors.

### 3.5. Physical Activity (IPAQ and PASE)

The IPAQ questionnaire also revealed no statistically significant differences in physical activity levels across the same sociodemographic and clinical characteristics: age (*p* = 0.500), BMI (*p* = 0.925), education (*p* = 0.072), employment (*p* = 0.123), smoking (*p* = 0.794), menstruation (*p* = 0.142), ABI right (*p* = 0.287), ABI left (*p* = 0.470), systolic blood pressure (*p* = 0.144) and diastolic blood pressure (*p* = 0.374).

However, the PASE questionnaire, which specifically evaluates activity among older participants, showed a statistically significant difference by education level (*p* = 0.024), indicating that women with higher education were more likely to report higher physical activity. This is consistent with previous findings that educational attainment positively correlates with activity levels in older adults.

### 3.6. Menstruation and Vascular Health

Analysis of menstruation status in relation to vascular parameters revealed that women who were still menstruating had significantly more favorable ABI values on the left side (*p* = 0.046), with 98.3% presenting normal results. This suggests a protective vascular effect during the reproductive years. By contrast, smoking was not significantly associated with ABI measurements (right ABI *p* = 0.383; left ABI *p* = 0.668), indicating no direct impact of smoking status on the ABI in this cross-sectional sample.

### 3.7. Association Between ABI and Lifestyle Factors

The relationship between ABI and lifestyle factors is summarized in Table 6. No significant associations were observed between ABI values and adherence to the Mediterranean diet (MEDAS) or physical activity levels as measured by the IPAQ and PASE questionnaires. Similarly, smoking status was not significantly associated with ABI. Only menstruation status showed a statistically significant relationship with ABI on the left side (*p* = 0.046), indicating better vascular function among women of reproductive age.

### 3.8. County-Level Findings

Further stratified analyses revealed additional regional differences.

In Primorje-Gorski Kotar County, low adherence to the Mediterranean diet was significantly more common among women with lower education (*p* = 0.026). PASE analysis showed that obesity was more frequent among less physically active participants (*p* = 0.013) and that reduced activity was associated with higher systolic blood pressure (*p* = 0.021). Importantly, all menstruating women in this county had normal ABI values (*p* = 0.032), reinforcing the association between reproductive status and vascular health. No significant associations were found between smoking and ABI results (right ABI *p* = 0.701; left ABI *p* = 0.275).

In Istria County, higher adherence to the Mediterranean diet was associated with several factors: older age (*p* = 0.022), postmenopausal status (*p* = 0.035), and retirement (*p* = 0.003). Additionally, participants with higher MEDAS scores tended to have lower systolic (*p* = 0.022) and diastolic blood pressure (*p* = 0.020), suggesting a cardioprotective effect of dietary habits in this region. Educational level also influenced physical activity in Istria, where more highly educated women demonstrated greater activity according to PASE results (*p* = 0.040). In contrast, menstruation status (right ABI *p* = 0.555; left ABI *p* = 0.372) and smoking (right ABI *p* = 0.124; left ABI *p* = 0.219) were not associated with ABI measurements in this subgroup.

### 3.9. Multivariable Analysis of Independent Predictors of Abnormal ABI

Multivariable logistic regression was conducted to identify independent predictors of peripheral arterial impairment after adjustment for potential confounding factors (age, body mass index [BMI], arterial hypertension, smoking, and county). Menopausal status was included as the main exposure of interest in all models.

Initially, the composite outcome of any ABI abnormality, defined as ABI < 1.00 in either leg (including borderline, mildly reduced, and pathological values), was analyzed. Among 438 women, 20 (4.6%) had ABI < 1.00. In the adjusted model, age emerged as a strong and statistically significant predictor of abnormal ABI: each additional year of age increased the odds by approximately 17% (aOR = 1.17 per year, 95% CI 1.09–1.26, *p* < 0.001). Current smoking was associated with higher odds of abnormal ABI (aOR = 3.56, 95% CI 0.97–13.11, *p* = 0.056), although this association did not reach conventional statistical significance; the wide confidence interval remained compatible with a clinically relevant effect. Hypertension showed a positive but non-significant association (aOR = 1.99, 95% CI 0.72–5.54, *p* = 0.186), while BMI was not independently related to ABI status (aOR = 1.07 per kg/m^2^, 95% CI 0.96–1.19, *p* = 0.225).

Postmenopausal status was associated with a higher crude prevalence of abnormal ABI, but this effect was attenuated after adjustment for age and other covariates (aOR = 0.10, 95% CI 0.01–1.03, *p* = 0.053). The confidence interval was wide and included both potentially protective and adverse effects, reflecting limited precision rather than evidence of no association. Women from Istria County tended to have lower odds of abnormal ABI compared with those from Primorje–Gorski Kotar County, but the difference was not statistically significant after adjustment (aOR = 0.40, 95% CI 0.14–1.14, *p* = 0.085).

When the outcome was restricted to definite PAD (ABI < 0.90 in either leg), only eight cases were identified (≈1.8% of the total sample). Due to the very small number of events relative to the number of covariates, the multivariable model for this stricter PAD definition showed quasi-separation, leading to unstable coefficient estimates and wide, unreliable confidence intervals. Consequently, adjusted odds ratios for ABI < 0.90 should not be interpreted quantitatively. This reflects limited statistical power rather than the absence of true associations.

Overall, the results indicate that older age is a robust and independent predictor of lower-limb arterial impairment in women, even after controlling for BMI, hypertension, and smoking. Smoking and hypertension showed consistent, directionally positive relationships with reduced ABI, although not statistically significant in this dataset. The difference between pre- and postmenopausal women diminished after adjustment for age, suggesting that part of the observed risk among postmenopausal women is mediated by chronological age and cumulative exposure to cardiovascular risk factors. Nevertheless, given the small number of women with markedly reduced ABI (<0.90), moderate effects of menopausal status or lifestyle cannot be ruled out.

The study had limited statistical power to detect associations for rare PAD outcomes. With fewer than 10 PAD cases among 438 participants, statistical power exceeded 80% only for large effect sizes (odds ratios ≈3 or greater) for common exposures such as hypertension or smoking, but was insufficient to detect smaller, yet clinically meaningful, effects (OR 1.5–2.0). Accordingly, all non-significant associations should be interpreted as inconclusive rather than truly null.

Table 7 presents the results of the multivariable logistic regression analysis for factors associated with an abnormal ankle–brachial index (ABI < 1.00). Increasing age remained the strongest independent predictor, while smoking and hypertension showed positive, though not statistically significant, associations after adjustment for confounding factors. Postmenopausal status lost significance after controlling for age, and county differences were attenuated.

When the outcome was restricted to definite peripheral arterial disease (PAD, ABI < 0.90), the number of cases was too small to permit reliable estimation of adjusted effects. As summarized in Table 8, the model suffered from quasi-separation due to the very low number of PAD cases (N = 8), and adjusted odds ratios are therefore not presented. These findings emphasize the limited statistical power for rare PAD outcomes in this cohort. Quasi-complete separation occurs when the number of outcome events is too small relative to the number of predictors, causing the regression algorithm to produce unstable or infinite coefficient estimates. In such cases, statistical results should be interpreted descriptively rather than inferentially.

A total of 8 cases (1.8%) met the PAD criterion (ABI < 0.90).

The multivariable model could not be reliably estimated due to quasi-separation caused by the very low number of events relative to covariates. As a result, adjusted coefficients and confidence intervals were unstable and are not presented.

This indicates limited statistical power rather than the absence of true associations.

## 4. Discussion

This study aimed to explore the relationships between cardiovascular health indicators, lifestyle factors, and sociodemographic characteristics among women from two coastal Croatian counties.

This study analyzed sociodemographic and health characteristics with emphasis on cardiovascular health, dietary habits, and physical activity. Associations between BMI, adherence to the Mediterranean diet, physical activity levels, cardiovascular system parameters, and sociodemographic factors were investigated. Special attention was given to differences between women of reproductive age and postmenopausal women. Additionally, the results were analyzed separately for Primorje–Gorski Kotar County and Istria County to explore potential regional variations.

The findings showed that nearly half of the total sample (47.1%) belonged to the age group of 61 years and older, indicating that the data were substantially influenced by an aging population. Older individuals are naturally more prone to chronic conditions, including hypertension and reduced vascular elasticity.

Educational level showed significant associations with several variables. Participants aged over 70 years with higher education were more likely to be physically active. Among highly educated participants, high physical activity was recorded in 25.0%, according to the PASE questionnaire. As physical activity protects against PAD and hypertension, these findings underline the need for educational interventions targeting older and less educated women. Murtagh et al. conducted a study among older adults in Ireland using the PASE questionnaire and found that adults with secondary or higher education were less likely to be classified as inactive compared with those without tertiary education (OR 0.79 for women, 0.89 for men) [29]. Vagetti et al. reported that older women with completed secondary education were 1.4 times more likely to meet the criteria for high physical activity compared to their peers with incomplete primary education [30]. Previous research has also shown that a higher level of education doubles the likelihood of engaging in sports or exercise, although it was not associated with greater activity in walking or gardening [31]. Interestingly, Shaw and Spokane found that educational attainment modifies the relationship between age and physical activity, with differences in activity levels across education groups increasing with age [32]. Collectively, these results indicate that different intervention strategies may be needed depending on the educational background of the older population.

In contrast, in Primorje–Gorski Kotar County, low adherence to the Mediterranean diet was observed among highly educated women in 53.8% of cases. This could be explained by the fact that women with higher education are often career-oriented and have less free time, leading to less time for preparing nutrient-rich meals compared to women with lower educational attainment. Consequently, this results in lower adherence to the Mediterranean diet. However, Kocaadam-Bozkurt et al. reported a positive association between educational level and adherence to the Mediterranean diet, which contradicts these findings [32].

Overall, 45.5% of respondents were retired. In Istria County, retirees demonstrated greater adherence to the Mediterranean diet compared to employed participants. This may be attributed to retirees having more free time for cooking healthy meals and, considering the local environment, continuing to use traditional, healthier ingredients. Navarro-Martinez et al. conducted a study in Valencia and found that adherence to the Mediterranean diet was not associated with age [33].

The results revealed that the majority of participants had normal ABI values (ABI right: 95.7%; ABI left: 95.0%), while mild to moderate PAD was present in 3.0–3.2% of participants, and severe PAD in 0.5–1.1%.

One of the key findings of the study was a significant association between menopause and cardiovascular health, particularly with ABI on the left side (*p* = 0.046). Normal ABI values were found in 98.3% of women of reproductive age, while postmenopausal women showed a higher proportion of abnormal values. In Primorje–Gorski Kotar County, all women who were still menstruating had normal ABI values. Postmenopausal women showed a higher prevalence of mild to moderate PAD, which may indicate the loss of the protective effect of estrogen, essential for vascular elasticity and endothelial health. These findings confirm that postmenopausal status is a key risk factor for the development of vascular impairment, emphasizing the need for regular screenings and early preventive measures, including increased physical activity and a diet rich in omega-3 fatty acids and antioxidants.

BMI analysis revealed a high proportion of overweight (35.9%) and obese individuals (17.6%), while 44.9% had normal body weight. Participants with higher BMI were more likely to have elevated blood pressure, which is expected since excess body weight is a known risk factor for arterial hypertension. However, no direct association was found between BMI and ABI values. Overweight status did not necessarily imply worse vascular health, suggesting that other factors such as diet and genetics also play a role in the development of cardiovascular disease (CVD).

Results from the MEDAS questionnaire indicated that 17.2% of participants had low adherence to the Mediterranean diet, 59.0% moderate adherence, and 23.8% high adherence. The analysis did not demonstrate a significant relationship between adherence to the Mediterranean diet and ABI values, suggesting that diet alone may not determine vascular health and that genetics and other lifestyle habits could be influential. Interestingly, in Istria County, older participants (71+) had higher adherence to the Mediterranean diet, while women of reproductive age were less likely to follow it. Similar to the greater adherence among retirees, this may be due to older individuals having more time to prepare meals and relying on traditional local foods typical of the Istrian region. In Istria County, participants with higher adherence to the Mediterranean diet also had lower blood pressure values.

According to the IPAQ questionnaire, most participants belonged to the high physical activity category (48.9%), while 30.3% were moderately active and 20.7% were low active. Participants with lower physical activity in Primorje–Gorski Kotar County were more likely to have elevated blood pressure; however, no significant association was found between physical activity levels and ABI values. In Primorje–Gorski Kotar County, less physically active individuals, according to the PASE questionnaire, were more often obese. These results align with expectations.

Although the findings between Primorje–Gorski Kotar County and Istria County were relatively similar, some differences emerged. These regional findings may reflect differences in age composition, urban–rural living conditions, lifestyle patterns, and accessibility or utilization of preventive health services. Urban settings may provide easier access to screening, specialist referrals and health education, whereas rural populations may face geographic, transportation, and organizational barriers that influence cardiovascular risk trajectories. Such differences should be considered when designing region-specific preventive strategies.

Istria County had a slightly higher prevalence of PAD compared to Primorje–Gorski Kotar County, possibly related to the older average age of participants and different dietary patterns. Postmenopausal women in Istria County exhibited worse vascular parameters than those in Primorje–Gorski Kotar County, which could reflect differences in lifestyle and access to healthcare. In Istria County, most participants lived in rural areas and may visit physicians less frequently due to limited transportation and fewer primary care facilities. Both systolic and diastolic blood pressure were higher in Istria County, likely due to older age distribution and lifestyle factors such as diet and physical activity. Smoking prevalence was significantly higher in Primorje–Gorski Kotar County (34.7%) compared to Istria County (20.5%). This finding may reflect differences between urban and rural lifestyles. Urban environments often have higher stress levels, more social interactions, and greater access to tobacco products, whereas rural settings may have a more conservative health approach and lower smoking rates. The analysis of IPAQ and PASE questionnaires showed no significant regional differences; however, older adults with higher education were generally more active.

The results suggest that menstruating women had better vascular indicators, with a higher percentage of normal blood pressure values. This association is likely due to the protective effects of estrogen, which plays a crucial role in maintaining cardiovascular and vascular health. These findings highlight the importance of targeted preventive programs for postmenopausal women to reduce CVD risk.

In the adjusted analyses, age emerged as the dominant factor associated with reduced ankle–brachial index, confirming the strong age-related decline in peripheral arterial perfusion among women. This finding is consistent with the progressive nature of arterial stiffening and atherosclerotic change that accompanies aging. Smoking and hypertension also showed directionally positive associations with lower ABI values, although these did not reach statistical significance, likely due to limited statistical power and the small number of abnormal ABI cases. The attenuation of the menopausal effect after adjustment for age suggests that the observed difference between pre- and postmenopausal women may be largely mediated by chronological aging rather than hormonal status alone. This finding raises the question of whether chronological aging, cumulative vascular exposure, and risk-factor duration outweigh the isolated hormonal transition. Menopause-related estrogen decline may accelerate endothelial dysfunction, while chronological aging contributes to arterial stiffening, oxidative stress, and micro-inflammation. Future research combining vascular imaging, hormonal profiling, and biomarkers of biological aging (e.g., arterial stiffness indices, inflammatory markers or vascular age scores) could help differentiate biological from chronological pathways. Differences between the two counties diminished after covariate adjustment, indicating that regional variability in ABI is explained primarily by differences in the age structure and cardiovascular risk profiles of the participants.

Analyses using ABI < 0.90 were limited by very low PAD prevalence, preventing stable regression modelling. Consequently, adjusted associations for definite PAD should be interpreted with caution, as the lack of statistical significance more likely reflects insufficient power rather than the absence of true effects. Overall, the findings highlight the importance of age, blood pressure control, and smoking prevention in maintaining lower-extremity arterial health among women, while also underscoring the need for larger, adequately powered studies to confirm these associations and to explore the independent role of menopausal transition in peripheral arterial disease risk.

### Limitations and Future Directions

The study’s cross-sectional design limits causal interpretation, and the use of self-reported questionnaires introduces potential recall bias. Additionally, the very low prevalence of definite PAD in our sample (approximately 1.8%) substantially limited the statistical power of multivariable modeling. As a result, non-significant associations should be interpreted as inconclusive rather than true null findings. Future studies with larger PAD case numbers are required to enable more robust effect estimation and avoid sparse-data bias. The absence of statistically significant associations between Mediterranean diet adherence or physical activity and ABI may also reflect the limited number of abnormal ABI outcomes as well as potential misclassification due to self-reported lifestyle measures. Future studies should incorporate objective lifestyle metrics such as accelerometry, wearable devices, dietary biomarkers, and longitudinal follow-up to better detect dose-response associations. Given the low number of PAD cases and the limited power for multivariable analyses, the findings should be interpreted cautiously. Although the sample is representative, the results may have limited generalizability to other populations. Future research should employ longitudinal designs with larger, more diverse cohorts and explore additional biological, genetic, and psychosocial factors influencing the development and progression of peripheral arterial disease (PAD). Preventive programs focused on improving diet quality, increasing physical activity, and promoting other cardiovascular-protective behaviors are urgently needed to reduce PAD risk, particularly in at-risk populations.

## 5. Conclusions

The present study provides new insights into the relationships between peripheral arterial health, sociodemographic characteristics, and lifestyle factors among women from two coastal Croatian counties. The findings confirm that older age is the strongest independent determinant of reduced ankle–brachial index, while smoking and hypertension show positive but non-significant associations after adjustment for confounding factors. No significant associations were observed between ABI values and adherence to the Mediterranean diet or physical activity levels, likely reflecting the predominance of normal ABI values in the sample.

Postmenopausal women exhibited a higher prevalence of mild to moderate peripheral arterial impairment, but this association diminished after adjustment for age, suggesting that chronological aging rather than menopausal status per se explains most of the observed differences. The very low number of cases with ABI < 0.90 limited the statistical power to detect associations for definite PAD.

Overall, the results emphasize the importance of early screening and age-appropriate cardiovascular prevention strategies in women, particularly targeting smoking cessation and blood pressure control. Future studies with larger, longitudinal cohorts and hormonal profiling are warranted to further clarify the independent role of menopause and lifestyle factors in the development and progression of peripheral arterial disease.

## Figures and Tables

**Table 1:** The classification of ABI measurement results.

ABI Value	Interpretation
>1.4	Peripheral vascular disease (mediosclerosis)
1.0–1.4	Normal value
0.9–1.0	Borderline value
0.8–0.9	Mild PAD
0.5–0.8	Moderate PAD
<0.5	Severe PAD

**Table 2:** Comparison Based on the Observed Counties.

		County					
		PRIMORJE-GORSKI KOTAR COUNTY		ISTRIA COUNTY		Total	
		N	%	N	%	N	%
Age	0–29	19	11.0%	13	4.9%	32	7.3%
	30–40	32	18.5%	22	8.3%	54	12.4%
	41–50	43	24.9%	34	12.9%	77	17.6%
	51–60	34	19.7%	34	12.9%	68	15.6%
	61–70	24	13.9%	77	29.2%	101	23.1%
	≥71	21	12.1%	84	31.8%	105	24.0%
	Total	173	100.0%	264	100.0%	437	100.0%
BMI (kg/m^2^)	Underweight	6	3.5%	1	0.4%	7	1.6%
	Normal weight	90	52.0%	106	40.2%	196	44.9%
	Overweight	55	31.8%	102	38.6%	157	35.9%
	Obesity	22	12.7%	55	20.8%	77	17.6%
	Total	173	100.0%	264	100.0%	437	100.0%
Menstruation	yes	99	57.2%	73	27.7%	172	39.4%
	no	74	42.8%	191	72.3%	265	60.6%
	Total	173	100.0%	264	100.0%	437	100.0%
Education	Lower	9	5.2%	33	12.5%	42	9.6%
	Secondary	92	53.2%	143	54.2%	235	53.8%
	Higher	12	6.9%	49	18.6%	61	14.0%
	University	60	34.7%	39	14.8%	99	22.7%
	Total	173	100.0%	264	100.0%	437	100.0%
Employment Status	Employed	116	67.1%	87	33.0%	203	46.5%
	Retired	36	20.8%	163	61.7%	199	45.5%
	Unemployed	21	12.1%	14	5.3%	35	8.0%
	Total	173	100.0%	264	100.0%	437	100.0%
Place of Residence	Urban	173	100.0%	25	9.5%	198	45.3%
	Rural	0	0.0%	239	90.5%	239	54.7%
	Total	173	100.0%	264	100.0%	437	100.0%

**Table 3:** Total number of chronic diseases in Primorje-Gorski Kotar County and Istria County.

CHRONIC DISEASES	TOTAL
Endocrine, nutritional, and metabolic diseases	121
Diseases of the digestive system	5
Diseases of the circulatory system	103
Diseases of the respiratory system	8
Diseases of the nervous system	8
Mental disorders and behavioral disorders	7
Diseases of the genital-urinary system	8
Diseases of the musculoskeletal system and connective tissue	22
Diseases of the blood and blood-forming organs and certain disorders of the immune system	19
Neoplasms	10
Diseases of the skin and subcutaneous tissue	1
Diseases of the eye and adnexa	3

**Table 4:** Total number of medications in Primorje-Gorski Kotar County and Istria County.

MEDICATION	TOTAL
Medications for thyroid disease	52
Sex hormones and modulators of the genital system	3
Psycholeptics	9
Psychoanaleptics	6
Antidiabetics	28
Antiprotozoal drugs	3
Anti-inflammatory and anti-rheumatic drugs	5
Beta-adrenergic blockers	38
Corticosteroids	3
Analgesics	3
Drugs acting on the nervous system	3
Agents acting on the renin-angiotensin system	148
Calcium channel blockers	73
Drugs modifying lipids	92
Heart medications	4
Immunosuppressants	6
Antidiarrheals	2
Diuretics	44
Antithrombotics	8
Vitamins	14
Antacids, ulcer disease medications, drugs affecting peristalsis	10
Medications for obstructive airway diseases	2
Minerals	5

**Table 5:** Data on Smoking, ABI, Blood Pressure, and Lifestyle Habits by County.

		County					
		PRIMORJE-GORSKI KOTAR COUNTY		ISTRIA COUNTY		Total	
		N	%	N	%	N	%
Smoking	Yes	60	34.7%	54	20.5%	114	26.1%
	No	113	65.3%	210	79.5%	323	73.9%
	Total	173	100.0%	264	100.0%	437	100.0%
ABI right	Severe PAD	0	0.0%	2	0.8%	2	0.5%
	Moderate PAD	0	0.0%	1	0.4%	1	0.2%
	Mild PAD	3	1.7%	3	1.1%	6	1.4%
	Borderline Value	2	1.2%	5	1.9%	7	1.6%
	Normal Value	166	96.0%	252	95.5%	418	95.7%
	Peripheral Vascular Disease (Mediaclerosis)	2	1.2%	1	0.4%	3	0.7%
	Total	173	100.0%	264	100.0%	437	100.0%
ABI left	Severe PAD	0	0.0%	5	1.9%	5	1.1%
	Moderate PAD	0	0.0%	0	0.0%	0	0.0%
	Mild PAD	1	0.6%	1	0.4%	2	0.5%
	Borderline Value	4	2.3%	7	2.7%	11	2.5%
	Normal Value	168	97.1%	247	93.6%	415	95.0%
	Peripheral Vascular Disease (Mediaclerosis)	0	0.0%	4	1.5%	4	0.9%
	Total	173	100.0%	264	100.0%	437	100.0%
Systolic BP (mmHg)	Normal Level	77	44.5%	71	26.9%	148	33.9%
	Elevated Blood Pressure	68	39.3%	116	43.9%	184	42.1%
	Hypertension	28	16.2%	77	29.2%	105	24.0%
	Total	173	100.0%	264	100.0%	437	100.0%
Diastolic BP (mmHg)	Normal Level	40	23.1%	34	12.9%	74	16.9%
	Elevated Blood Pressure	120	69.4%	200	75.8%	320	73.2%
	Hypertension	13	7.5%	30	11.4%	43	9.8%
	Total	173	100.0%	264	100.0%	437	100.0%
MEDAS	Low Compliance	26	15.0%	49	18.6%	75	17.2%
	Moderate Compliance	106	61.3%	152	57.6%	258	59.0%
	High Compliance	41	23.7%	63	23.9%	104	23.8%
	Total	173	100.0%	264	100.0%	437	100.0%
IPAQ	Low Activity	26	17.2%	41	23.8%	67	20.7%
	Moderate Activity	50	33.1%	48	27.9%	98	30.3%
	High Activity	75	49.7%	83	48.3%	158	48.9%
	Total	151	100.0%	172	100.0%	323	100.0%
PASE	Low Activity	13	59.1%	47	51.1%	60	52.6%
	Moderate Activity	5	22.7%	29	31.5%	34	29.8%
	High Activity	4	18.2%	16	17.4%	20	17.5%
	Total	22	100.0%	92	100.0%	114	100.0%

**Table 6:** Association between ankle–brachial index (ABI) and lifestyle factors (dietary adherence and physical activity).

Variable	ABI Right—*p* Value	ABI Left—*p* Value	Significance
**MEDAS (Mediterranean diet adherence)**	0.867	0.634	n.s.
**IPAQ (physical activity < 70 yrs)**	0.287	0.470	n.s.
**PASE (physical activity ≥ 70 yrs)**	—	—	n.s.
**Smoking**	0.383	0.668	n.s.
**Menstruation status**	—	0.046	*significant*

(n.s. = not significant).

**Table 7:** Multivariable logistic regression analysis of independent predictors of abnormal ankle–brachial index (ABI < 1.00) among women from Primorje–Gorski Kotar and Istria Counties.

Variable	Adjusted OR (aOR)	95% Confidence Interval	*p*-Value
**Age (per year)**	**1.17**	**1.09–1.26**	**<0.001**
**Body mass index (per kg/m^2^)**	1.07	0.96–1.19	0.225
**Arterial hypertension (yes vs no)**	1.99	0.72–5.54	0.186
**Current smoking (yes vs. no)**	3.56	0.97–13.11	0.056
**Postmenopausal status (vs. reproductive age)**	0.10	0.01–1.03	0.053
**County (Istria vs. Primorje–Gorski Kotar)**	0.40	0.14–1.14	0.085

Model statistics: n = 438 women; cases with ABI < 1.00 = 20 (4.6%). Nagelkerke R^2^ = 0.32; Hosmer–Lemeshow *p* = 0.41 (adequate fit). Dependent variable = abnormal ABI (<1.00 in either leg).

**Table 8:** Multivariable logistic regression for predictors of definite peripheral arterial disease (PAD, ABI < 0.90).

Variable	Adjusted OR (aOR)	95% Confidence Interval	*p*-Value
**Age (per year)**	–	–	–
**Body mass index (per kg/m^2^)**	–	–	–
**Arterial hypertension (yes vs. no)**	–	–	–
**Current smoking (yes vs. no)**	–	–	–
**Postmenopausal status (vs. reproductive age)**	–	–	–
**County (Istria vs. Primorje–Gorski Kotar)**	–	–	–

## Data Availability

Further information concerning the present study is available from the corresponding authors upon reasonable formal request.
